# Neural Responses to Multimodal Ostensive Signals in 5-Month-Old Infants

**DOI:** 10.1371/journal.pone.0072360

**Published:** 2013-08-19

**Authors:** Eugenio Parise, Gergely Csibra

**Affiliations:** Cognitive Development Center, Central European University, Budapest; University of Turin and the Italian Institute of Technology, Italy

## Abstract

Infants' sensitivity to ostensive signals, such as direct eye contact and infant-directed speech, is well documented in the literature. We investigated how infants interpret such signals by assessing common processing mechanisms devoted to them and by measuring neural responses to their compounds. In Experiment 1, we found that ostensive signals from different modalities display overlapping electrophysiological activity in 5-month-old infants, suggesting that these signals share neural processing mechanisms independently of their modality. In Experiment 2, we found that the activation to ostensive signals from different modalities is not additive to each other, but rather reflects the presence of ostension in either stimulus stream. These data support the thesis that ostensive signals obligatorily indicate to young infants that communication is directed to them.

## Introduction

Communicative signals ('ostensive signals', [1]) function to indicate communicative intent and to specify the addressee of this intent. They can occur in different forms and in different modalities. Eye-contact is a visual signal, calling someone's name or using special intonation patterns (to make the addressee know that she is the intended recipient of some message) are auditory stimuli, and there are even amodal signals, like contingent responsivity that can carry ostensive content [1]. What makes these stimuli similar is that they attract the same interpretation (the recipient feels being addressed) and that they generate similar expectations (of some communicative content from the same source).

There is plenty of evidence that young infants, and even newborns, display special sensitivity to stimuli that adults consider ostensive signals. For example, newborns prefer to look at faces with direct gaze compared to averted gaze [2], and prefer to listen to infant-directed speech compared to adult-directed speech [3]–[5]. By 5 month of age, they learn to extract gaze direction from faces that are not oriented directly to them [6], and consecutive facial signals modulate each other's effect on the brain activation of infants [7]. Around the same age, infants can already extract infant-directed intonation patterns from background noise [8], become attuned to the specific level of contingency that indicate that someone is interacting with them (e.g. [9]), and start to learn new ostensive signals, such as their name [10].

While all these findings are consistent with the proposal that infants interpret these stimuli as ostensive signals, they do not confirm this hypothesis directly. One way to test this proposal is to investigate whether infants expect to receive further communication upon detecting ostensive signals, which they should do if they interpret these stimuli as indicating the presence of a message directed to them. Some findings suggest that they do so: infants are more likely to follow someone's gaze after eye contact, infant-directed speech, and contingent reactivity than in the absence of these signals [11], [12]. Furthermore they pay special attention to objects after hearing their own name [13] as indicated by the infant’s attention-sensitive Negative Central (Nc) ERP component (for detailed description and source localization of the Nc component see [14], [15]).

Another way to test the hypothesis that all these stimuli are interpreted as ostensive signals is to check whether infants treat these stimuli as equivalent to each other. Behaviourally, this seems to be the case, as infants respond similarly to these signals: by paying more attention to, and by smiling at, the source [1]. However, these responses may be based on different neural mechanisms and may indicate analogous reactions to ostensive signals rather than being the manifestations of the same underlying representation. In adults, evidence suggests that ostensive signals from different modalities influence each other's perception [16] and activate common brain regions. For example, Kampe, Frith, and Frith [17] found similar neural responses to direct gaze (eye contact) and the participants name ('John, hey, John') in the medial prefrontal cortex (MPFC) and the temporal poles. The same brain regions are also activated in response to interpreting communicative intentions not directly addressed to the participants [18], [19]. However, the MPFC responds much stronger when one is feeling being the target of social interaction initiated by someone else [20].

Studies with infants also tend to find frontal activation in response to ostensive stimuli. Grossmann, Johnson, Farroni, and Csibra [21] reported gamma-band (∼40 Hz) oscillation to direct vs. averted gaze over orbito-frontal areas in 4-month-old infants. Similar activations could also be recorded in response to dynamic gaze shifts that result in eye-contact with the viewer, and haemodynamic measurements confirmed the origin of this activation in the prefrontal cortex [7]. Frontal activation can also be measured in newborns in response to prosodic speech [22] and infant-directed speech [23] by near-infrared spectroscopy (NIRS). In older infants, frontal responses to infant-directed speech are modulated by the familiarity of the voice [24]. It has been shown by electrophysiological measures that words uttered in infant-directed intonation processed differently from adult-directed words, though this difference was found only for familiar words in 6-month-olds [25].

We know only one study that directly contrasted the neural activation to ostensive signals of different modalities in infants. Grossmann, Parise, and Friederici [26] reported that both eye contact and hearing their own name produced prefrontal activation in 5-month-old infants (measured by NIRS). Although these effect did not overlap, they originated from adjacent brain regions and were correlated across modalities. In the present study, we attempted to find common electrophysiological indices of brain activation to eye contact and infant-directed speech. The existence of such indices would support the proposal that these stimuli are interpreted the same way – as ostensive signals. In addition, electrophysiological measures, unlike haemodynamic activation measure by NIRS, could also indicate whether such interpretation occurs early or late in the processing of the stimuli.

The second aim of our study was to investigate the nature of the response that ostensive signals elicit by combining stimuli from different modalities. One can advance three different hypotheses about the effects of such combinations depending on the cognitive mechanisms that are reflected in these activations. First, if the effect of ostensive signals is simply the amplification of non-specific arousal or attention in the infant, the combination of the eliciting stimuli would result in an additive effect: the more ostension, the higher activation. For example, the Nc component is known to be sensitive to manipulations influencing infants' attention [14]. If this hypothesis is correct, we should find an increase of Nc in response to an ostensive signal, and an additive increase on this component in the presence of a combination of such signals. The second possibility is that the response is obligatory to any ostensive signal, and it is not modulated by additional stimuli, even if they are relevant for assessing the presence of communicative intention. This hypothesis predicts an OR relation: both eye contact and infant-directed speech will generate the response, but their combination is not different from the effect of either. According the third hypothesis, a non-ostensive signal in one modality (e.g., no eye contact) would be treated as evidence of absence of communicative intention, and would cancel the effect of an ostensive signal (e.g., infant-directed speech) in the other modality. This hypothesis predicts an AND relation between concurrent stimuli: only the combination would be treated as sufficient evidence of communicative intention.

We developed a paradigm to test these hypotheses in two experiments measuring event-related potentials (ERPs) and gamma-band event-related oscillations. Experiment 1 looked for signs of shared activation between a visual ostensive signal (direct gaze, as opposed to averted gaze) and an auditory one (infant-directed vs. adult-directed speech). Experiment 2 combined these signals into multimodal stimuli to test the nature of the mechanisms that process them. The data of both experiments are available upon request.

## Experiment 1

Five-month-old infants watched a static female face with closed eyes on a computer screen while they were exposed four types of transient stimuli: eye opening with direct gaze, eye opening with averted gaze, a pseudo-word in infant-directed speech, or the same word in adult-directed intonation. We measured their EEG to investigate common activation to ostensive signals in the two modalities, contrasted with non-ostensive control stimuli. We predicted that prefrontal gamma-band oscillations would display the interpretation of ostensive stimuli as communicative signals in both modalities.

### Methods

#### Ethics statement

The parents of all participants provided written informed consent, and this study was approved by the United Ethical Review Committee for Research in Psychology (EPKEB) at Central European University.

#### Participants

Eighteen infants participated in the study (9 females; average age = 148.17 days, range = 136 to 157 days). Thirteen additional infants were excluded because of fussiness (n = 3), insufficient number of trials (n = 9), technical problems or experimenter error (n = 1). The minimum inclusion criterion was artifact-free EEG recording in at least 10 trials within each experimental condition. All infants were born full term (gestational age: 37 to 41 weeks) and in the normal weight range (>2500 g).

#### Experimental design

We applied four within-subject experimental conditions, corresponding to the orthogonal crossing of the factors of Modality (visual vs. auditory) and Ostension (ostensive vs. non-ostensive). In this design, we contrasted the ostensive visual stimulus of direct gaze (DG) with the non-ostensive visual stimulus of averted gaze (AG), and the ostensive auditory stimulus of infant-directed speech (IDS) to the non-ostensive auditory stimulus of adult-directed speech (ADS).

#### Stimuli

A female face (size 15.5×9.5 cm) with closed eyes was constantly presented on the monitor on a black background. The visual stimulus events were produced by replacing this face with other versions of the face in which the eyes were open, revealing the iris either in the middle (direct gaze, DG) or at the right or left corner (averted gaze, AG). One eye covered a surface of about 2×0.9 cm and the distance between the two eyes was 4.6 cm. The eyebrows in the open-eye images were raised by about 0.5 cm compared to the image with closed eyes.

The auditory stimulus was a pseudo-word, “Toda” pronounced by a female voice with two different intonation: either infant- or adult-directed-speech (IDS and ADS, respectively). The recording of the two words were digitized at 32 bit resolution and 48 kHz sampling rate, and were edited with Audacity (v. 1.2.5) and Praat (v. 5.1). The words had the equal length of 1000 ms, and the duration of the first syllable was about 290 ms. The average volume intensity was 61.86 dB for the IDS and 61.50 dB for the ADS stimulus.

#### Apparatus

Visual stimuli were presented on a 19-inch CRT monitor operating at 100 Hz refresh rate using PsychToolBox (v. 3.0.8) and custom-made Matlab® scripts. Auditory stimuli were presented by a pair of computer speakers located behind the monitor. A remote control video camera located below the monitor allowed the recording of infants' behaviour during the experiment.

High-density EEG was recorded continuously using Hydrocel Geodesic Sensor Nets (Electrical Geodesics Inc., Eugene, OR, USA) at 124 scalp locations referenced to the vertex (Cz). The ground electrode was at the rear of the head (between Cz and Pz). Electrophysiological signals were acquired at the sampling rate of 500 Hz by an Electrical Geodesics Inc. amplifier with a band-pass filter of 0.1–200 Hz.

#### Procedure

Infants sat on their parent lap 70 cm from the CRT monitor. At the beginning of each trial, a dynamic attention grabber (a small dynamic visual stimulus) appeared on top of the face, between the eyes, for 600 ms. Then the attention grabber stopped moving, and the display remained frozen for an interval randomly varying between 600 and 800 ms. Then attention grabber disappeared and a visual (DG or AG) or auditory (IDS or ADS) stimulus was presented for 1000 ms. Visual stimuli with open eyes were immediately followed by the image with closed eyes. An inter-trial interval between 1100 and 1300 ms was inserted between successive trials, while the face with closed eyes remained on the screen. Infants were presented with a maximum of 192 trials divided into 4 blocks. Trials were presented equiprobably in pseudo-random order with the following constraints: no more than two consecutive trials of the same modality in a row; no more than three consecutive trials of the same ostensive value in a row. Trials were presented as long as the infants were attentive. If they became fussy, the experimenters gave a short break to them. The session ended when the infants' attention could no longer be attracted to the screen. The behaviour of the infants was video-recorded throughout the session for off-line trial-by-trial editing.

#### EEG analysis

The digitized EEG was band-pass filtered between 0.3–100 Hz and was segmented into epochs including 500 ms before stimulus onset and 1500 ms following stimulus onset for each trial. EEG epochs were automatically rejected for body and eye movements whenever the average amplitude of a 80 ms gliding window exceeded 55 µV at horizontal EOG channels or 200 µV at any other channel. Additional rejection of bad recording was performed by visual inspection of each individual epoch. Bad channels were interpolated in epochs in which ≤10% of the channels contained artifacts; epochs in which >10% of the channels contained artifacts were rejected. Infants contributed on average 12.11 artifact free trials to the DG condition (range: 10 to 19), 11.67 to the AG condition (10 to 15), 11.67 to the IDS condition (10 to 19), 12.61 to the ADS condition (10 to 22).

The artifact free segments were subjected to time-frequency analysis to uncover stimulus-induced oscillatory responses. The epochs were imported into Matlab® using the free toolbox EEGLAB (v. 9.0.5.6b) and re-referenced to average reference. Using a custom-made scripts collection named ‘WTools’ (available at request), we computed complex Morlet wavelets for the frequencies 10–90 Hz with 1 Hz resolution. We calculated total-induced oscillations performing a continuous wavelet transformation of all the epochs by means of convolution with each wavelet and taking the absolute value (i.e., the amplitude, not the power) of the results (see [27]). Transformed epochs were then averaged for each condition separately. To remove the distortion introduced by the convolution, we chopped 300 ms at each edge of the epochs, resulting in 1400 ms long segments, including 200 ms before and 1200 ms after stimulus onset. We used the average amplitude of the 200 ms pre-stimulus window as baseline, subtracting it from the whole epoch at each frequency.

On the same artifact free segments, averaged event-related potentials (ERPs) were calculated separately for each stimulus condition. The ERPs were baseline-corrected with respect to the average amplitude in the 200 ms window preceding stimulus onset, and were re-referenced to the average reference.

### Results

#### Induced gamma-band responses

**Figure 1:**
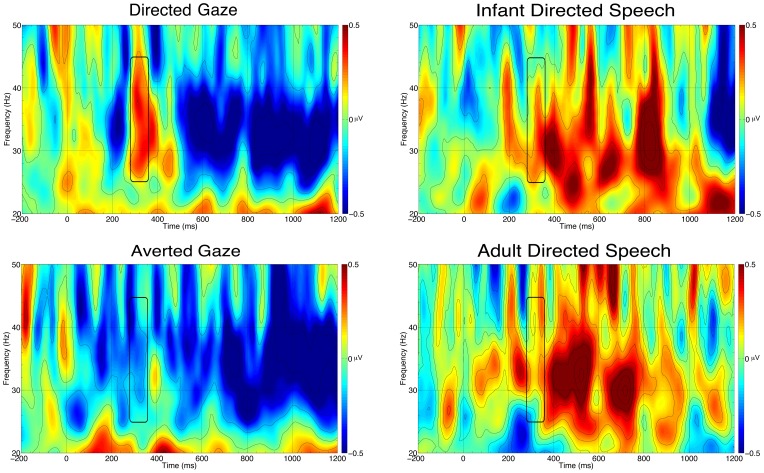
Time frequency plots for each condition in Experiment 1. Each plot is the average of the analyzed channels on the frontal area. The black rectangle marks the analyzed time window and frequency band.

Based on previous results [7], [21], we selected the scalp area, time window and frequency band, and measured the induced gamma-band activity over the forehead (the average of channels 9, 15, and 22, corresponding to Fp1, Fpz, and Fp2, respectively) in the 280 to 360 ms time window, and 25 to 45 Hz frequency window (Figure 1). An ANOVA with Modality (visual vs. auditory) and Ostension (ostensive vs. non-ostensive) as within-subject factors revealed a main effect of Ostension: *F*(1,17) = 6.75, *p* = .019, *η^2^_p_* = .28, with ostensive stimuli eliciting stronger gamma synchronization. Collapsing the data across modalities, 13 of 18 subjects displayed this effect (Wilcoxon’s *Z* = −2.24, *p* = .03). Separate comparisons of gamma-band activation against baseline in each condition yielded a significant effect only for direct gaze (DG): *t*(17) = 2.07, *p* = .05.

#### Event-related potentials

**Figure 2:**
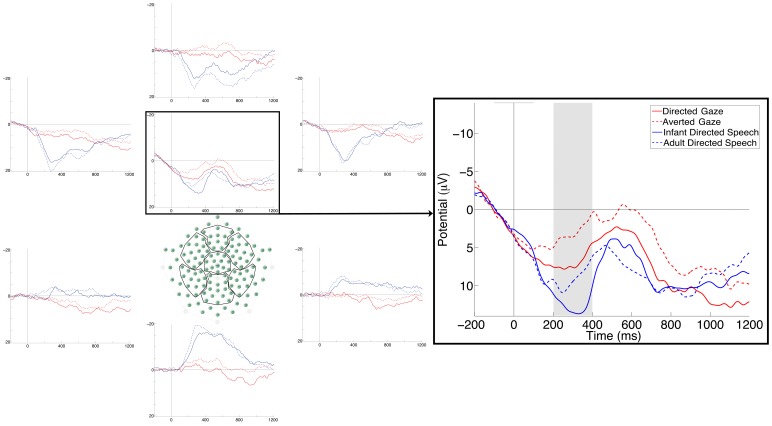
Average ERPs at 7 scalp regions (left), and ERPs at all analyzed channels over the central area of the scalp (right) in Experiment 1. The grey rectangle marks the analyzed time window.

Because we did not have any specific hypothesis concerning ERP effects of ostension, we visually inspected the grand averages to find a component that displayed similar effects of ostension in both modalities. We identified such a positive component peaking about 300 ms post-stimulus around the vertex bilaterally (see Figure 2). This component was elicited by both auditory and visual stimuli, and was more positive to ostensive than to non-ostensive trials in both the visual (DG vs. AG) and the auditory (IDS vs. ADS) modality. We quantified this component by measuring the average amplitude between 200 and 400 ms in three ROIs: on channels 7, 13, 30, 31, and 37 (roughly corresponding to the area between C3 and Cz in the 10–20 international system), on channels 6, 55, and Cz (central midline), and on channels 80, 87, 105, 106, 112 (the area between C4 and Cz). To confirm the effect, we performed an ANOVA on these data with Modality, Ostension and ROI (left vs. central vs. right) as within-subjects factors. (Note that this analysis cannot be considered a hypothesis testing but rather an exploratory statistic). The ANOVA revealed a main effect of Ostension (*F*(1,17) = 8.27, *p* = .01, *η^2^_p_* = .33), with ostensive stimuli eliciting a higher amplitude compared to non-ostensive ones, independently from modality. Collapsing the data across modalities, 13 of 18 subjects showed the effect (Wilcoxon’s *Z* = −2.46, p = .01). (Eight infants displayed the effect of Ostension in both oscillatory and ERP measures.) We also found a main effect of Modality (*F*(1,17) = 7.45, *p* = .01, *η^2^_p_* = .31), with auditory stimuli eliciting higher amplitude compared to visual ones. The ANOVA revealed a main effect of ROI as well (*F*(2,34) = 8.65, *p* = .001, *η^2^_p_* = .34) with the amplitude at left electrodes higher than at both central and right lateralized electrodes (Bonferroni post hoc test: *p* = .002 and *p* = .006 respectively). Finally, we found an interaction between Modality and ROI (*F*(2,34) = 6.30, *p* = .005, *η^2^_p_* = .27), because the amplitude of auditory stimuli at left channels was significantly higher than the amplitude of auditory stimuli at central and right channels, while there was no ROI effect in the responses to visual stimuli (Bonferroni post hoc test: all ps <.00006; only the comparisons between auditory-central and auditory-right, and the comparisons of the visual stimuli with each other were not significant).

On the same channels we analyzed the Nc component measuring the average amplitude between 400 and 700 ms in the same three ROIs. An ANOVA with the same between subjects factors (Ostension, Modality and ROI) revealed a main effect of modality (*F*(1,17) = 5.85, *p* = .03, *η^2^_p_* = .25) with visual stimuli more negative than auditory stimuli. We also found a main effect of ROI (*F*(2,34) = 6.26, *p* = .005, *η^2^_p_* = .27), with the amplitude at right channels more negative than at left channels (Bonferroni post hoc test: *p* = .004). Finally we found an interaction between Modality and ROI *(F*(2,34) = 5.20, *p* = .011, *η^2^_p_* = .23), because the amplitude of auditory stimuli on the left and central channels was significantly lower than the amplitude to visual stimuli on left, central and right channels; moreover the amplitude of auditory stimuli on the right channels was significantly higher than the amplitude of visual stimuli at central channels and significantly lower than the amplitude of auditory stimuli at left and central channels.

We also tested whether we managed to replicate a previously reported effect of infant-directed speech in infants of similar age [25]. Such an effect was visible in the grand average over temporal sites (channels 39, 45 and 50 around T3 on the left hemisphere; channels 101, 108 and 115 around T4 on the right hemisphere) at around 350 ms. We quantified the difference by computing the average amplitude across these channels between 250 and 500 ms. An ANOVA with Speech (IDS vs. ADS) and ROI (left vs. right) as within subjects factors revealed only a main effect of Speech (*F*(1,17) = 6.20, p = .02, *η^2^_p_* = .27), being IDS more negative than ADS. A similar analysis on the same channels, with Gaze instead of Speech as within subjects factor, did not lead to any significant result (all *ps* >.1), suggesting that this effect was modality specific.

### Discussion

Frontal gamma-band oscillations in Experiment 1 replicated those of Grossmann et al. [21], despite the fact that our stimuli were considerably different. Instead of flashing faces with direct or averted gaze in front of infants, we exposed them to a more natural visual change (eye opening), and found a similar brain activation (see also [7]). Note that this activation was fast (started around ∼300 ms after eye opening) and short (see Figure 1). Our statistical analysis suggested that a similar effect was also present in response to a word in infant-directed speech, at least when it was compared to adult-directed speech (not to baseline). However, this effect did not emerge as a result of activation in the IDS and non-activation in the ADS condition. Rather, it seems that the same frontal circuit was activated by both speech stimuli, but this activation started earlier for infant-directed than for adult-direct speech (Figure 1). This pattern of results suggests that there may be common mechanisms underlying the recognition of ostensive signals in different modalities, and it contributes to the interpretation of the stimuli from the outset rather than being the conclusion of lengthy perceptual processing.

We also identified a potential signal sensitive to the ostensive nature of the stimuli in the ERPs. This effect also occurred early, concurrently with the gamma-band activation. Since this effect was not predicted in advance, we remain cautious about its interpretation, as it could also be a fluke. However, the main point of Experiment 1 was to identify effects with potential functional significance for the processing of ostensive signals in order to use them in Experiment 2 to assess neural responses to compounds. At first sight, our ERP finding appears at odd with the finding of Zangl and Mills [25], who found a negativity related to the processing of familiar words with infant-directed intonation in 6-month-olds. Their effect was localized to left temporal sites, which we were able to replicate in the early, but not in the late time window (600–800 ms post-stimulus), and, unlike in the original study, it occurred in response to unfamiliar (novel) words. Note also that Zangl and Mills did not report results from midline electrodes, where we identified an effect sensitive to the ostensive nature of stimuli in both auditory and visual modality.

The analysis of the later time window, where the attention-sensitive Nc component should occur [15] found no effect of ostension. This suggests that the effect of ostensive stimuli is not mediated by general attention mechanisms, and cannot be attributed to the increase of arousal.

## Experiment 2

Having identified potential signatures of the processing mechanisms of ostensive signals in Experiment 1, we turned to the question of the nature of these mechanisms. In particular, Experiment 2 investigated whether multimodal compound signals generate an additive effect of the unimodal signatures of the recognition of communication or they interact in a special way.

### Methods

#### Participants

Eighteen infants participated in the study (7 females; average age = 140.44 days, range = 123 to 152 days). Twenty-four additional infants were excluded because of fussiness (n = 11), insufficient number of trials (n = 9), technical problems or experimenter error (n = 2), poor impedance (n = 1), or not matching the selection criteria (n = 1, this infant was identified as a preterm after participation). We applied the same inclusion criteria as in Experiment 1. Note that the Ethic Statement declared for Experiment 1 applies to Experiment 2 as well.

#### Experimental design

This study also included four within-subject experimental conditions, but now all conditions were audio-visual. Thus, the two orthogonal factors were Gaze (ostensive DG vs. non-ostensive AG) and Speech (ostensive IDS vs. non-ostensive ADS). In this design, we contrasted a bimodally ostensive stimulus (DG+IDS) to unimodally ostensive stimuli (DG+ADS and AG+IDS) and to a non-ostensive compound (AG+ADS).

#### Apparatus and stimuli

The same apparatus and stimuli were used as in Experiment 1.

#### Procedure

The procedure were similar to that of Experiment 1, except that each trial included both a visual stimulus (eye opening) and an auditory one (a word). The four types of trials were presented equiprobably in pseudo-random order with the following constraints: no more than two consecutive equal auditory stimuli in a row; no more than three consecutive equal visual stimuli in a row.

#### EEG analysis

The data was analyzed the same way as in Experiment 1. Infants contributed on average 13.83 artifact free trials to the DG+IDS condition (range: 10 to 27), 14.06 to the DG+ADS condition (10 to 22), 14.56 to the AG+IDS condition (10 to 31), 13.72 to the ADS condition (10 to 27).

### Results

#### Induced gamma-band responses

**Figure 3:**
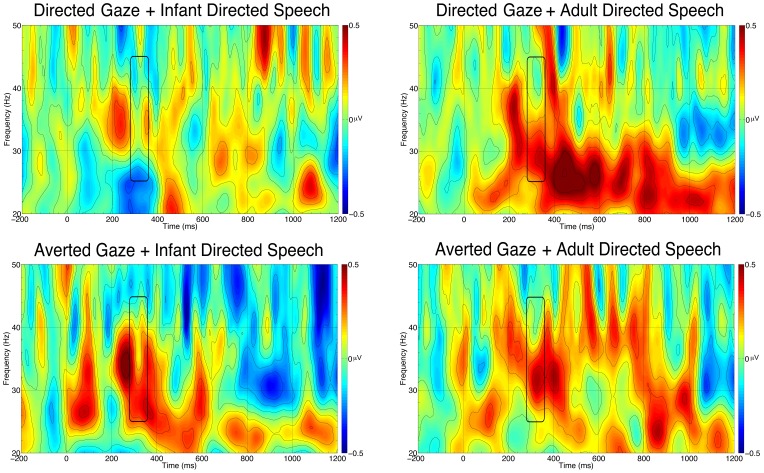
Time frequency plots for each condition in Experiment 2. Each plot is the average of the analyzed channels on the frontal area. The black rectangle marks the analyzed time window and frequency band.

We calculated the average amount of induced gamma-band responses in the four conditions the same way as we did in Experiment 1. An ANOVA with Gaze (ostensive vs. non-ostensive) and Speech (ostensive vs. non-ostensive) as within-subject factors revealed no significant main effects or interactions (Figure 3). Separate comparisons of gamma-band activity against the baseline yielded no significant effect in any condition (Figure 3).

#### Event-related potentials

**Figure 4:**
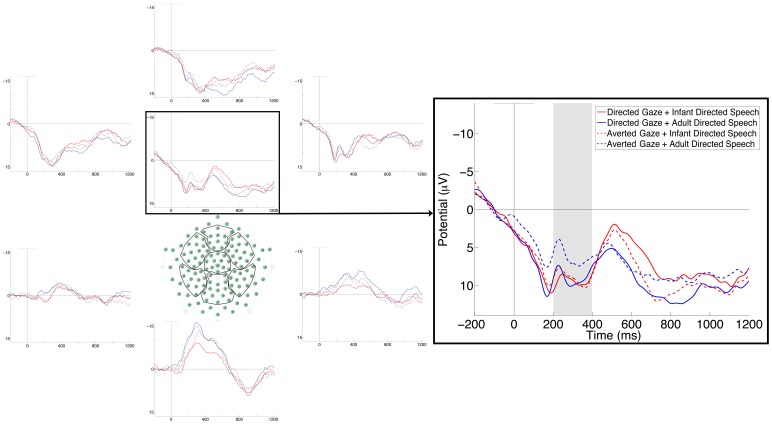
Average ERPs at 7 scalp regions (left), and ERPs at all the analyzed channels on the central area of the scalp (right) in Experiment 2. The grey rectangle marks the analyzed time window.

To test whether the effect of ostensive stimuli in different modalities were additive, we quantified the communication-sensitive component identified in Experiment 1 in the four conditions in the present study (Figure 4). We then performed a three-way ANOVA with Gaze, Speech and ROI (left vs. central vs. right) as within-subject factors, which yielded a three-way interaction (*F*(2,34) = 4.57, *p* = .017, *η^2^_p_* = .21). Bonferroni post-hoc tests revealed that the non-ostensive AG+ADS stimulus produced significantly less positive response than all other conditions in the central and right ROIs (all *ps* <.05). The post-hoc comparison also revealed that DG+IDS at left channels elicited significantly higher amplitude than did DG+IDS at central, AG+IDS at right, and AG+ADS at left channels (all ps <.04). We also found a main effect of ROI (*F*(2,34) = 8.92, *p* = .001, *η^2^_p_* = .34), with the amplitude at left electrodes higher than at both central and right electrodes (Bonferroni post hoc test: *p* = .004 and *p* = .002, respectively).

We also analyzed the Nc component on the same channels and in the same time window we used in Experiment 1. The ANOVA revealed a main effect of ROI (*F*(2,34) = 4.58, *p* = .017, *η^2^_p_* = .21). Post hoc comparisons revealed that the amplitude of the channels on the right was significantly lower than the amplitude of the channels on the left (*p* = .02). We also found a three-way interaction (*F*(2,34) = 5.13, *p* = .011, *η^2^_p_* = .23). Bonferroni post hoc test revealed that on the left channels the ostensive DG+IDS stimulus was more negative than the non-ostensive AG+ADS stimulus (*p* = .04). We also found that the ostensive DG+IDS stimulus on the central and right channels was more negative than the mixed stimulus DG+ADS on the left and central channels and of the non-ostensive stimulus AG+ADS on the left channels (all *ps* <.004). Finally we found that the mixed stimulus AG+IDS on the right channels and the non-ostensive stimulus AG+ADS at central and right channels were more negative than the non-ostensive stimulus AG+ADS at left channels (all *ps* <.04).

### Discussion

Unexpectedly, the combination of ostensive signals from different modalities eliminated, rather than strengthened, the prefrontal gamma-band response in 5-month-olds. Although some oscillations were evident on the time-frequency maps (Figure 3), statistically they did not differ either from baseline or from each other. A possible explanation for this result is that the original effect [21], which we replicated and extended in Experiment 1, does not reflect a cerebral response to ostensive stimuli but is an artefact. For example, if direct gaze elicits more microsaccades than does averted gaze in 5-month-olds [28], but concurrent speech (whether infant- or adult-directed) inhibits such a response, microsaccade-related gamma-band activity would only be expected in response to unimodal eye-contact stimuli. In the absence of high-resolution eye-movement recording in our study, we are unable to confirm or disconfirm such an account, though the fact that we did find a weak prefrontal gamma-band response to infant-directed speech in Experiment 1 may speak against it. Gamma-band oscillatory responses can be elusive and difficult to localize [29], which may have also contributed to the absence of this response in our recordings.

In contrast, the ERP responses that we identified in Experiment 1 were found again in Experiment 2, and produced interpretable results. This response did not display additivity across the two modalities, suggesting that the effect of ostensive signals cannot be reduced to increasing a quantitative aspect of their processing (e.g., facilitating attention to them). Rather, the combined effect of visual and auditory ostensive signals (compared to their non-ostensive counterparts) was the same as that of either of them alone. Such pattern of results indicates that, for 5-month-old infants, a stimulus is either ostensive or not, but cannot be 'more ostensive' than another stimulus. This response seems to be obligatory, as it occurred even when only one modality delivered an ostensive signal. Thus, eye-contact produced the response even if the intonation of the accompanying speech did not indicate that the infant was the addressee, and infant-directed speech was also effective when the only face in front of the infants did not look at them. Together with the short latency of this effect, the obligatory nature of the response is consistent with the proposal that it represents an early stage of stimulus processing rather than being the result of effortful integration of stimuli of different modalities.

Note, however, that such integration might occur later on during stimulus processing, as it is suggested by the analysis of the Nc component. The three-way interaction and the complex pattern of the post-hoc effects we found are not sufficient to clarify whether modulation of the Nc reflects an effort for cross-modal integration of ambiguous stimuli. This question has to be addressed in further studies.

## General Discussion

We addressed the question whether infants' well-documented sensitivity and attention to certain social signals reflects the interpretation of these stimuli as indicating ostensive communication directed to them. We approached this question by comparing (Experiment 1) and combining (Experiment 2) communicative stimuli from two modalities. We found that, just like in adults [17], these stimuli produce overlapping neural activation for 5-month-old infants in two different measures (Experiment 1). This result allowed us to draw two kinds of conclusions. First, whatever the neural mechanisms underlying these activation reflect, the processes that are sensitive to the interpretation of the ostensive signals rather than some low level stimulus feature. This is because the stimuli in question (direct gaze and infant-directed speech) share no common physical parameters, especially when they are contrasted with their non-ostensive counterparts (averted gaze and adult-directed speech). Thus, the processing of ostensive signals, whatever stimulus modality they represent, is channelled to the same neural mechanisms, probably in the frontal cortex, as suggested by previous literature (see [7], [17], [26]). Second, both oscillatory and evoked responses to ostension emerged very early in the time course of processing, at about 300 ms after stimulus onset. Considering that cortical responses to faces [30] and infant-directed prosody [25] can be recorded with about the same latency at this age, this early activation suggests a preferential treatment of ostensive signals.

The nature of this fast and modality-independent process was addressed in Experiment 2. We considered three competing hypotheses (see Introduction). If the common activation to direct gaze and infant-directed speech reflects increased attention (or other non-specific mechanism) induced by these ostensive stimuli, one would expect that the combination of these signals produces even higher activation than a unimodal stimulus. We did not find evidence for such an additive mechanism. Alternatively, if the stimuli from the two modalities are integrated into a single signal, one may expect that the non-ostensive nature of one component (e.g., averted gaze) would cancel the interpretation of the other stimulus (e.g., infant-directed speech) as an ostensive signal ('She may speak to another infant'). We did not find evidence for such a mature integration of multimodal stimuli either. Rather, the combined stimuli elicited the same activation as either of them, confirming the hypothesis that the neural activation to these signals represent a rigid and obligatory response. (This conclusion is also strengthened by the early latency of the response.) The most plausible interpretation of this response is that it manifests the fast and rudimentary interpretation of the eliciting stimuli as ostensive signals, i.e., as indicating the presence of a communicative intention targeting the infant [1].

We wish to remain cautious in speculating about the precise neural mechanisms, and about the brain substrates, of these responses. This is partly because our data were not as strong as we had expected: we did not replicate the gamma-band oscillatory response to ostensive stimuli in Experiment 2, and our interpretation relied on a component post-hoc identified in Experiment 1. Moreover we investigated only two types of ostensive stimuli, and so our findings might apply to mutual gaze and IDS only. Nevertheless, we see no reason to refraining from giving a functional interpretation of this ERP response in terms of reflecting the processing of ostensive signals. Further research will have to clarify which further stimuli, if any, will activate the same processes and what brain regions and neural computations are manifested in the ERP component we identified here.

Our results also raise developmental questions concerning the interpretation of ostensive signals. Five-month-old infants did not produce differential activation to a bimodally ostensive stimulus (DG+IDS, fully ostensive and not contradictory signal) and to unimodally ostensive stimuli (DG+ADS and AG+IDS, only partially ostensive and contradictory signals). Future research should further investigate whether older infants learn to inhibit the early automatic response to ostensive signals by canceling the extra attention paid to the stimulus in one modality if its interpretation is not corroborated by the accompanying signal from another modality. Such inhibition would allow infant a more accurate selection of consistent vs. inconsistent sources of communication, looking for communicative partners rather than particular combination of signals.

### Conclusions

Human communication, whether it is verbal or non-verbal, is ostensive - it makes manifest that the source has a communicative intention. We found that 5-month-old infants process the signals that convey this manifestation the same way independently from the modality in which it is expressed, suggesting that they are sensitive to ostension as such. The neural activations correlated with such processing indicate that the response to ostensive signals is obligatory at this age, probably reflecting a rudimentary interpretation of these signals as indicators of communication addressed to the infant and triggering the ensuing search for communicative content from the same source.
